# Reconstructing Historical VOC Concentrations in Drinking Water for Epidemiological Studies at a U.S. Military Base: Summary of Results

**DOI:** 10.3390/w8100449

**Published:** 2016-10-13

**Authors:** Morris L. Maslia, Mustafa M. Aral, Perri Z. Ruckart, Frank J. Bove

**Affiliations:** 1 Agency for Toxic Substances and Disease Registry, Atlanta, GA 30341, USA; 2 Multimedia Environmental Simulations Laboratory, School of Civil and Environmental Engineering, Georgia Institute of Technology, Atlanta, GA 30332, USA

**Keywords:** historical reconstruction, modeling, drinking water, water quality, volatile organic compounds (VOC), epidemiological study, health risk, Camp Lejeune

## Abstract

A U.S. government health agency conducted epidemiological studies to evaluate whether exposures to drinking water contaminated with volatile organic compounds (VOC) at U.S. Marine Corps Base Camp Lejeune, North Carolina, were associated with increased health risks to children and adults. These health studies required knowledge of contaminant concentrations in drinking water—at monthly intervals—delivered to family housing, barracks, and other facilities within the study area. Because concentration data were limited or unavailable during much of the period of contamination (1950s–1985), the historical reconstruction process was used to quantify estimates of monthly mean contaminant-specific concentrations. This paper integrates many efforts, reports, and papers into a synthesis of the overall approach to, and results from, a drinking-water historical reconstruction study. Results show that at the Tarawa Terrace water treatment plant (WTP) reconstructed (simulated) tetrachloroethylene (PCE) concentrations reached a maximum monthly average value of 183 micrograms per liter (μg/L) compared to a one-time maximum measured value of 215 μg/L and exceeded the U.S. Environmental Protection Agency’s current maximum contaminant level (MCL) of 5 μg/L during the period November 1957–February 1987. At the Hadnot Point WTP, reconstructed trichloroethylene (TCE) concentrations reached a maximum monthly average value of 783 μg/L compared to a one-time maximum measured value of 1400 μg/L during the period August 1953–December 1984. The Hadnot Point WTP also provided contaminated drinking water to the Holcomb Boulevard housing area continuously prior to June 1972, when the Holcomb Boulevard WTP came on line (maximum reconstructed TCE concentration of 32 μg/L) and intermittently during the period June 1972–February 1985 (maximum reconstructed TCE concentration of 66 μg/L). Applying the historical reconstruction process to quantify contaminant-specific monthly drinking-water concentrations is advantageous for epidemiological studies when compared to using the classical exposed versus unexposed approach.

## 1. Introduction

**Figure 1:**
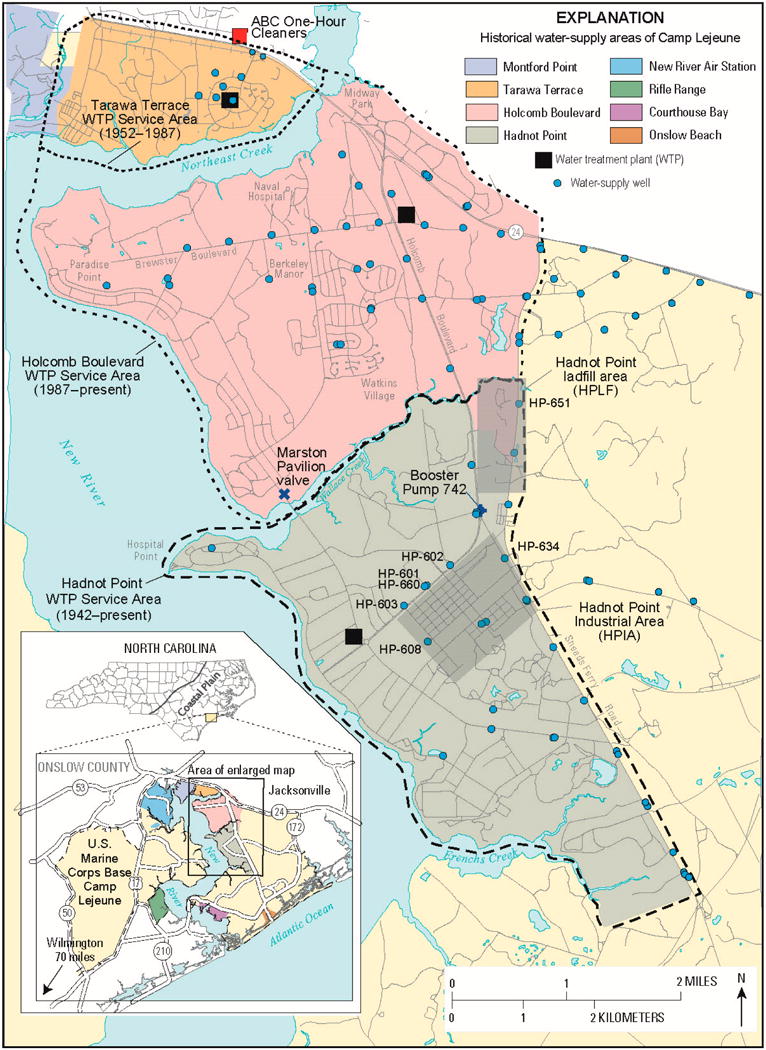
Water-supply areas with focus on housing areas, barracks, and workplaces included in the Agency for Toxic Substances and Disease Registry (ATSDR) drinking-water exposure and health studies, U.S. Marine Corps Base Camp Lejeune, North Carolina (modified from [1]; 1 mi = 1.61 km).

The Agency for Toxic Substances and Disease Registry (ATSDR) conducted epidemiological studies to evaluate health risks in children and adults because of exposures to drinking water contaminated with volatile organic compounds (VOCs) at U.S. Marine Corps Base (USMCB) Camp Lejeune, North Carolina (Figure 1). VOCs of interest to the ATSDR studies were tetrachloroethylene (PCE), trichloroethylene (TCE), *trans*-1,2-dichloroethylene (1,2-tDCE), vinyl chloride (VC), and benzene [1] (Table A3). Measured water-quality data and simulated result are discuss ed in terms of the U.S. Environmental Protection Agency’s (USEPA) maximum contaminant levels (MCLs) Sor the afore mentioned VOCs: 100 micrograms per liter (μg/L) for 1,2-tDCE, 5 μg/L for PCE, TCE, and benzene, and 2 μg/L for VC. Refer to [2,3] for properties, drinking-water standards, regulations, and MCLs related to VOCs and other chemicals.

Many years of effort have gone into ATSDR’s drinking-water exposure and health studies at USMCB Camp Lejeune resulting in numerous agency reports and published papers. Owing to brevity, this paper integrates these efforts, reports, and papers into a synthesis of the overall approach to, and results from, the drinking-water historical reconstruction study. With respect to the three housing areas, barracks, and workplaces of interest to the ATSDR drinking-water exposure and health studies—Tarawa Terrace, Hadnot Point, and Holcomb Boulevard (Figure 1)—Tarawa Terrace results have been previously published [4,5] and will solely be summarized herein. Approaches, methods, and results for the Hadnot Point and Holcomb Boulevard housing areas are presented for the first time herein in the peer-reviewed scientific literature. Specific details for the Tarawa Terrace, Hadnot Point, and Holcomb Boulevard drinking-water analyses are also presented in publicly available ATSDR reports [1,4].

The ATSDR epidemiological studies required estimates or direct knowledge of contaminant-specific concentrations in drinking water—at monthly intervals—delivered to housing, barracks, and workplaces within the study areas. Because of limited or unavailable historical drinking-water concentration data during much of the health-study period (January 1968–December 1985), the historical reconstruction process, which included substantial efforts in information gathering and data mining, water-modeling methods, and sensitivity and probabilistic analyses were used to estimate monthly mean contaminant-specific concentrations. These methods and analyses included linking materials mass balance (mixing) and water-distribution system models to groundwater-flow and contaminant fate and transport models to derive and quantify monthly mean concentrations and ranges of concentrations for contaminants of interest to the ATSDR epidemiological studies (PCE, TCE, 1,2-tDCE, VC, and benzene). TCE, VC, and benzene are classified as carcinogenic to humans and PCE is classified as probably carcinogenic to humans [6,7].

## 2. Materials and Methods

### 2.1. Background

USMCB Camp Lejeune is located in the Coastal Plain of North Carolina, in Onslow County, southeast of the City of Jacksonville and about 70 miles (113 km) northeast of the City of Wilmington, North Carolina. In general, the study area is bounded to the north by North Carolina Highway 24 (SR 24), to the west by New River, to the south by Frenchs Creek, and generally to the east by the drainage divides of upstream tributaries of Wallace and Frenchs Creeks (Figure 1). Northeast Creek separates the Tarawa Terrace base housing area from the Hadnot Point and Holcomb Boulevard base housing areas. Operations began at USMCB Camp Lejeune during late 1941 [8] with the Hadnot Point water treatment plant (WTP) coming on line during 1942 and servicing the entire base until other WTPs were constructed and brought online, such as the Tarawa Terrace WTP during 1952–1953 and the Holcomb Boulevard WTP during June 1972 (Table S1). In 1989, USMCB Camp Lejeune and ABC One-Hour Cleaners (Figure 1) were placed on the USEPA’s National Priorities List (NPL) of hazardous waste sites. A chronological list of selected events related to water supply and environmental contamination at USMCB Camp Lejeune and vicinity is provided in Table S1.

### 2.2. Water Supply and Conctamination

Groundwater is the sole source of water supply for USMCB Camp Lejeune. Eight water-distribution systems have supplied or currently (2016) supply drinking water to family housing, barracks, workplaces, and other facilities at USMCB Camp Lejeune. The three water-distribution systems of interest to the ATSDR health studies—Tarawa Terrace, Hadnot Point, and Holcomb Boulevard (Figure 1)—have historically supplied drinking water to the majority of family housing units, enlisted personnel barracks, and workplaces at the base. ATSDR documented information and aggregated data related to water-supply chronology within the study areas of USMCB Camp Lejeune. Details pertinent to water-supply well operations (e.g., construction, in-service, and out-of-service dates) and WTP operations are provided in [1,4,9,10].

Hadnot Point was the original water-distribution system, serving the entire base with drinking water beginning in the early 1940s. The Hadnot Point WTP was constructed and began operations likely during 1941–1942. The Tarawa Terrace WTP began delivering drinking water during 1952–1953, and the Holcomb Boulevard WTP began delivering drinking water during June 1972 (Table S1). Currently (2016), the Hadnot Point WTP services the Hadnot Point area, and the Holcomb Boulevard WTP services the Holcomb Boulevard and Tarawa Terrace base housing areas because the Tarawa Terrace WTP was shut down during 1987 due to contamination of several supply wells [4,5].

The Holcomb Boulevard water-distribution system is connected to the Hadnot Point water-distribution system at the Marston Pavilion valve and at booster pump 742 (Figure 1). Booster pump 742 was removed during 2007, but the two systems can still be interconnected by opening a valve at the same location. For operational reasons, the two water-distribution systems are rarely connected—exceptions being some documented (and undocumented) intermittent connections that occurred during late spring and summer months of 1972–1986 and a continuous 8-day period of 28 January–4 February 1985 [1,4,11] (refer to Camp Lejeune water documents (CLW) 6774–8761, 8109, and 8117 in [11]). Additional discussion of the aforementioned interconnection periods is provided in this paper in the Section 3.2 and in [1]. Operational chronologies for water-supply wells in the Hadnot Point and Holcomb Boulevard study areas during the period 1942–2008 are provided in [10,12] and shown in Figure S1. The graph shows dates of operation for each well that supplied raw water to the Hadnot Point and Holcomb Boulevard WTPs, the dates when some of the wells were permanently taken out of service, and wells with documented contamination. Water-supply well operations for Tarawa Terrace WTP service area are provided in [4].

During the early 1980s, high concentrations of VOCs were discovered in groundwater and drinking water serving some areas at USMCB Camp Lejeune. Within the Hadnot Point WTP service area, groundwater was contaminated mostly with TCE, as well as PCE and refined petroleum products, such as benzene, toluene, ethylbenzene, and xylene (BTEX). Historical base operations and lack of environmentally protective disposal practices at USMCB Camp Lejeune have been identified as being responsible for contamination of groundwater and drinking-water supplies within the Hadnot Point WTP service area [9,13]. Within the Holcomb Boulevard WTP service area, drinking water remained predominantly uncontaminated except for intermittent supply during spring and summer months by contaminated Hadnot Point water during years 1972–1985. Within the Tarawa Terrace WTP service area, groundwater was contaminated mostly with PCE. An off-base dry-cleaning facility (ABC One-Hour Dry Cleaners—Figure 1) was identified as being responsible for contaminating several on-base water-supply wells at Tarawa Terrace [4,5,14]. Maximum measured concentrations of selected contaminants within the study areas have been documented as follows [1,4,9,11]:

- 1400 μg/L of TCE in treated drinking water at the Hadnot Point WTP (May 1982),
- 380 μg/L and 720 μg/L of benzene in a Hadnot Point WTP supply well (July and December 1984, respectively),
- 215 μg/L of PCE in treated drinking water at the Tarawa Terrace WTP (February 1985), and
- 1580 μg/L of PCE in a Tarawa Terrace WTP supply well (January 1985).

ATSDR is required to gather information and data to assess human health impacts from exposures at NPL sites. Because of the potential exposures to high VOC concentrations, ATSDR began health studies in 1995 to evaluate effects of exposure to contaminated drinking water.

### 2.3. Historical Reconstruction Methods

**Figure 2:**
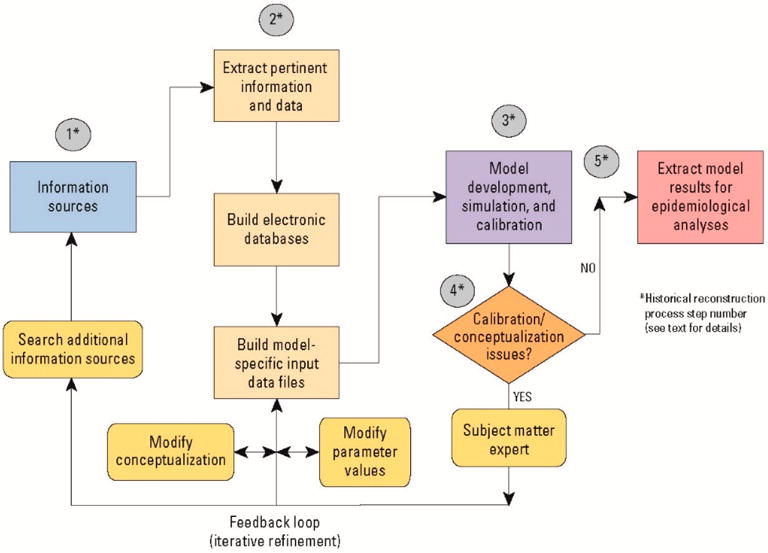
Generalized process of identifying information, extracting usable model-specific data, and applying models to reconstruct historical drinking-water contaminant-specific concentrations (from [1]).

When direct, past knowledge of contaminant concentrations in drinking water is limited or data are unavailable, historical reconstruction methods can be used to provide estimates of contaminant concentrations. Characteristically, historical reconstruction includes information gathering and data mining activities and the application of simulation tools, such as models, to re-create or represent past conditions [1,4,5,15–21]. For ATSDR’s drinking-water exposure analyses at USMCB Camp Lejeune, methods included linking materials mass balance (mixing) and water-distribution system models to groundwater-flow and contaminant fate and transport models [1,4,5]. Historical reconstruction results for contaminant-specific concentrations were needed at monthly intervals for the purposes of the epidemiological studies. That is because standard practice in epidemiological studies of adverse reproductive outcomes is anassessment of exposures (whether environmental, occupational, or diet risk; factors) at ehe monthly or trimester level [22] (pp. 602–603). Ideally, these analyses required monthly contaminant concentrations at water-supply wells and at the WTPs. The generalized five-step process used to identify information sources, extract usable model-specific data, and develop, apply, and calibrate models to reconstruct historical contaminant-specific concentrations in drinking water at USMCB Camp Lejeune is shown graphically in Figure 2. The five-steps of the process are: (1) review information sources; (2) extract information and data and develop databases; (3) develop, simulate, and calibrate models; (4) determine if model conceptualization or calibration issues exist, and if they do, use subject matter experts to iteratively refine model databases and search for additional information sources; end (5) assess when sufficient agreement exists between water-level and contaminant concentration data (historical and present-day) and model results. At that point, historical drinking-water concentration simulation results were extracted from model-output databases and provided to ATSDR epidemiologists foe use in the USMCB Camp Lejeune epidemiological analyses [23–27]. By its very nature, historical reconstruction is an iterative process (Figure 2). Contaminant-specific monthly mean concentrations obtained from information sources and data, water-modeling techniques, and the historical reconstruction process were used in the epidemiological studies to estimate the level and duration of exposures to the mother during her pregnancy, to the infant (up to 1 year of age), and to adults (e.g., navy and masine personnel and civilian employees of the Base). It is important to note that throughout the historical reconstruction process, data analysts and water modelers were blinded to the health outcome status of the individuals included in the epidemiological studies.

Substantial effort and resources were dedicated to the task of identifying information sources and extracting data because of the voluminous and disparate sources of information and data pertinent to the study area [1] (Appendix A2). The purpose was to obtain information and data that could be extracted and transformed into digital databases in order to conduct historical reconstruction analyses using a modeling approach. By its very nature, information discovery and data mining is not an exact process that can be used or relied upon to identify a single, specific piece of information or data point. Once pertinent model-specific data were identified and extracted, they were entered into digital databases. Model-specific input databases were then developed from these digital databases.

The analyses and simulation tools used as part of the historical reconstruction process included: (1) geohydrologic analyses; (2) water-distribution system field testing; (3) water-level data to characterize groundwater flow; (4) groundwater-flow and contaminant fate and transport models (for dissolved and light nonaqueous-phase liquid [LNAPL] constituents); (5) parameter sensitivity and uncertainty analyses; (6) probabilistic Markov analyses; and (7) water-distribution system modeling. Brief descriptions of each analysis and simulation tool, the type of analysis (e.g., data, interpretation, or simulation) and supporting references are listed in Table 1. Several novel and innovative methods and models were developed as part of the historical reconstruction process owing to the very complex character of the study area, the complex historical water-supply well operations (Figure S1), and the need to reconstruct monthly mean contaminant-specific concentrations. Summarized below are some of these novel and innovative methods:

- Effective and efficient (with respect to published methods) fire-flow test method for water-distribution system model calibration [28],
- Historical monthly operations and pumped groundwater volumes reconstructed for nearly 100 supply wells [29],
- Linear state-space representation of a contaminated aquifer developed to reconstruct historical concentrations in supply wells without the need to use traditional numerical fate and transport modeling [30],
- Volume estimates of lost benzene and LNAPL fate and transport in groundwater [31], and
- Probabilistic Markov process to estimate the number of intermittent transfers of drinking water between a contaminated and uncontaminated drinking-water system [12].

Specific details and descriptions for each type of analysis and each type of model or computational tool are provided in the in [1].

#### 2.3.1. Modeling of Groundwater Flow and Contaminant Fate and Transport

A three-dimensional groundwater-flow model was developed and calibrated based on interpretations of geohydrologic data [32], analyses and interpretations of water-level data, and the conceptual model of groundwater flow for the study area [33]. The groundwater-flow model of the study area consists of 7 layers that were correlated with geologic and hydrogeologic units. Model layers 1, 3, 5, and 7 are correlated with water-bearing units or aquifers, and corresponding model layers 2, 4, and 6 are correlated with semi-confining units or aquitards. Calibrated model parameter values used for simulating groundwater flow and contaminant fate and transport for the Hadnot Point and Holcomb Boulevard study areas are listed in Table S2. Detailed descriptions of deriving the calibrated model input parameter values and calibrated groundwater-flow model input files for use with the MODFLOW model code [34] are provided in [1].

Fate and transport simulations of contaminants of concern in groundwater were conducted for PCE, TCE, 1,2-tDCE, VC, and benzene. Owing to the physical and chemical properties of these contaminants and the rigorous analyses of available sampling data, a variety of simulation tools were applied. For example, the simulations of PCE and TCE were conducted as dissolved-phase constituents, whereas the simulation of benzene required volume estimates for floating product, simulation of benzene as a LNAPL, and simulation of benzene as a dissolved constituent in groundwater. Because of space limitations in this paper, groundwater fate and transport simulation details and results are presented solely for TCE. The focus on TCE is chosen because TCE is the primary contaminant in groundwater and drinking water within the Hadnot Point study area and its characteristics in groundwater are similar to those of PCE. Readers desiring complete details on the simulation of the fate and transport in groundwater of other contaminants should refer to the publicly available ATSDR reports [1,31,36].

#### 2.3.2. Modeling of Drinking-Water Concentrations

Because all water-supply wells were mixed at the Hadnot Point WTP prior to treatment and distribution a materials mass balance (mixing) model, which is based on the principles of continuity and conservation of mass [42], was used compute monthly mean concentrations of TCE in drinking water delivered to base housing and other facilities during the period 1942–1985. Application of the mixing model presumes that the computed concentrations of TCE in drinking water at the WTP are nearly equal to the TCE concentrations of drinking water at any location throughout the WTP service area. This approach was used in [4,5] to reconstruct historical drinking-water concentrations for the Tarawa Terrace study. Applying the mixing model approach to successfully and accurately represent historical water-distribution system contaminant concentrations is documented in [46].

During the period June 1972–December 1985, the Hadnot Point and Holcomb Boulevard water-distribution systems were intermittently interconnected during dry spring and summer months and for an eight-day period of 28 January–4 February 1985. During these periods, contaminated Hadnot Point drinking water was transferred to and distributed within the uncontaminated Holcomb Boulevard water-distribution system. The interconnection of the two water-distribution systems was primarily accomplished by operating booster pump 742, although on rare occasions, the valve at Marston Pavilion (near Wallace Creek) also was opened (Figure 1). Operational records indicating booster pump 742 operations and Marston Pavilion valve openings were only partially documented. Interconnection information and data were obtained from the USMCB Camp Lejeune water utility log books [11] (CLW 7023–8735).

A more complex analysis was necessary (compared to the simple mixing-model approach) to determine the concentration of drinking water in the Holcomb Boulevard water-distribution system during periods of interconnection of the Hadnot Point and Holcomb Boulevard water-distribution systems. This required the application of the EPANET 2 water-distribution system model [45] and extended period simulation (EPS). The EPANET 2 water-distribution system model was calibrated for the Holcomb Boulevard water-distribution system using field data collected by ATSDR; field data represented operational conditions during 2004 [12,28,47,48]. EPSs were used to reconstruct water-distribution system flow and mass transport patterns during discrete interconnection events when booster pump 742 was intermittently operated, resulting in the transfer of contaminated drinking water from the Hadnot Point water-distribution system to the uncontaminated Holcomb Boulevard water-distribution system. Pipelines represented in the water-distribution system network models are coincident with locations of streets within the Hadnot Point and Holcomb Boulevard area (Figure 1) (e.g., see [46] (Figure I3)). The network representation of the Hadnot Point and Holcomb Boulevard water-distribution systems was simplified by representing the Hadnot Point water-distribution system as an infinite reservoir on the upstream side of booster pump 742; this allowed for shorter EPANET 2 model runtimes. For the 8-day period, 28 January–4 February 1985, the bypass valve at Marston Pavilion (Figure 1) was documented to have been open, thereby allowing contaminated Hadnot Point drinking water to flow freely through the bypass valve to the Holcomb Boulevard water-distribution system. Because information pertaining to times when interconnection events occurred was limited, and for some years unknown (e.g., 1972–1977), a Markov process [44] was applied using available information to estimate the probability and number of monthly interconnection events that occurred during the months of April–August for years 1972–1985 [1,12].

## 3. Historical Reconstruction Analyses and Results

Summaries of results of the historical reconstruction analyses within the Hadnot Point and Holcomb Boulevard areas are discussed and presented in this section of this paper (see [4,5] for historical reconstruction analyses for Tarawa Terrace and vicinity). Results are presented for the following analyses: (1) simulation of three-dimensional groundwater flow; (2) simulations of the fate and transport of TCE within the Hadnot Point Industrial Area (HPIA) and within the Hadnot Point landfill (HPLF) area; (3) computation of monthly mean drinking water concentrations of PCE, TCE, 1,2-tDCE, VC, and benzene at the Hadnot Point WTP; and (4) reconstructed concentrations of TCE within the Holcomb Boulevard housing areas during periods of intermittent water transfers (1972–1985) from the Hadnot Point WTP to the Holcomb Boulevard WTP service area. Complete and detailed descriptions and discussions of historical reconstruction analyses and results are provided in [1,12,30,31,35,36].

### 3.1. Fate and Transport of Trichloroethylene (TCE) in Groundwater

A calibrated predevelopment water-level model-input database was a pre-requisite for conducting transient groundwater-flow and contaminant fate and transport simulations—specifically in the HPIA and HPLF areas. Predevelopment groundwater flow was conceptualized using approximately 773 water-level measurements [33,35]. A predevelopment (steady-state) potentiometric surface map of the Brewster Boulevard aquifer system for the study area was developed by using these water-level data along with stream-gage data, climatic data and the geohydrologic framework [1,32]. A potentiometric surface map showing water-level measurements that were used as control points, water-level contours, and the generalized directions of groundwater flow used as the basis for calibrating the predevelopment, three-dimensional groundwater-flow model for the Hadnot Point and Holcomb Boulevard areas are provided in [1,35].

The simulated predevelopment potentiometric contours for the Brewster Boulevard aquifer system, derived from simulated water levels, are shown Figure S2. The goodness of fit of the predevelopment calibration was assessed by calculating residuals between measured and simulated water levels [35]. Results of the residual analysis are also shown in Figure S2. Within the areas of interest for contaminant fate and transport (the HPIA and HPLF areas) the resulting residuals generally range within ±5 ft (±1.5 m). For the entire active model domain, nearly 90% of the residuals are within a range of ±5 ft (±1.5 m), which is indicative of an acceptable calibration. Also shown in Figure S2 are simulated directions of groundwater flow, which indicate groundwater originating in the highlands areas and discharging to streams, creeks, and the New River, and flowing through the HPIA and HPLF areas. Comparing the simulated directions of groundwater flow with the estimated directions of groundwater flow in [1] (Figure A16) indicates general agreement between model results and the conceptual model of groundwater flow for the Hadnot Point and Holcomb Boulevard study area.

Four TCE source locations were identified within the HPIA (Figure 1 and Figure S3a) from numerous potential contaminant sources for inclusion in contaminant fate and transport model simulations; two TCE sources were identified within the HPLF area for inclusion in contaminant fate and transport model simulations. The identification, documentation, timelines, locations, and references of contaminant sources are provided in [1] (Table A8). Specific details pertinent to representation of the TCE sources in the contaminant fate and transport model are presented in [36]. Table S3 lists the contaminant sources, locations, concentrations, and durations used for fate and transport simulations within the HPIA and HPLF area. For the HPIA, sources are located near the Building 900 area and near Buildings 1115, 1401, and 1601 (Figure S3a). For historical reconstruction and modeling purposes, it was assumed that TCE sources were introduced to the HPIA during January 1951 (Buildings 1115, 1401, and 1601) and January 1957 (Building 900 area); sources were removed from model simulations during June 1993 (Buildings 1115 and 1601), December 1993 (Building 1401), and December 1994 (Building 900 area). All contaminated water-supply wells were removed from service by December 1985 and were similarly accounted for during model simulations. For the HPLF area (Figure 1 and Figure S3b), well HP-651 was the primary water-supply well affected by groundwater contamination. For historical reconstruction and modeling purposes, the two TCE sources were introduced to the HPLF model during January 1948. Well HP-651 began operations during July 1972, was removed from service by February 1985 [10] and was similarly accounted for during model simulations.

**Figure 3:**
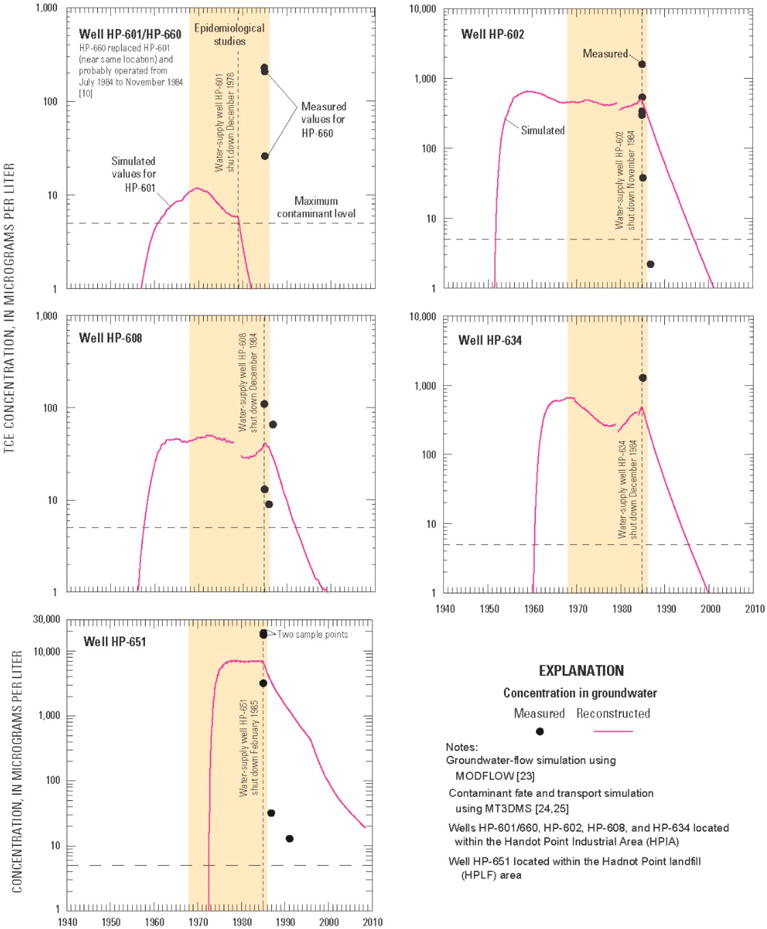
Reconstructed (simulated) and measured concentrations of trichloroethylene (TCE) at selected water-supply wells within the Hadnot Point Industrial Area and the Hadnot Point landfill area at U.S. Marine Corps Base Camp Lejeune, North Carolina (modified from [1]).

Figure 3 shows the reconstructed (simulated) TCE concentrations for water-supply wells HP-601/660, HP-602, HP-608, and HP-634 within the HPIA and HP-651 for the HPLF area. Note, water-supply well HP-660 replaced HP-601 and probably operated from July 1984 to December 1984. Monthly reconstructed TCE concentration results occur on the last day of the month (e.g., 31 January); they are interpreted as being representative of simulated values on any given day of that month. The results are designated and referred to herein as “monthly mean concentrations of TCE.” The reconstructed concentrations at water-supply wells are flow-weighted concentration values for supply wells that are open to multiple water-bearing units [35,36]. As can be seen in the graphs of Figure 3, observation data in water-supply wells are very limited and in some instances provide as few as one data point by which to compare reconstructed TCE concentrations (e.g., HP-634). For water-supply wells HP-602 and HP-608, measurements were taken 1 day apart or within a 1-month or less time span, whereas model results represent a mean concentration over an entire month. In the case of HP-651, three of the five water-quality samples were obtained between 16 January and 4 February 1985, and range from 3200 μg/L to 18,900 μg/L. This makes it difficult to uniquely calibrate a numerical model that at best only approximates the physics, chemistry, and biology of “real-world” conditions and relies on limited observation data, which are subject to measurement error. Given the aforementioned limitations and constraints, the reconstructed (simulated) TCE concentrations provide reasonable agreement with observed data and “real-world” conditions.

Areal distributions of reconstructed TCE concentrations for model layers 1, 3, and 5 within the HPIA for four periods—January 1951, January 1968, November 1984, and June 2008—are shown in Figure S4. Model layers 1, 3, and 5 represent major water-bearing units in the study area and are correlated with the Brewster Boulevard aquifer system, the Tarawa Terrace aquifer, and the Upper Castle Hayne aquifer, respectively [1] (Table A11). Water-supply wells in the study area were open to water-bearing units corresponding to model layers 3 and 5. The specific simulation dates noted above were selected to show typical historical reconstruction results because: (1) January 1951 represents an early time period after the onset of pumping; (2) January 1968 represents the start of the core period for the epidemiological studies; (3) November 1984 represents the month prior to the shutdown of many of the contaminated water-supply wells; and (4) June 2008 represents the end of the historical reconstruction simulation and a time when all contaminated water-supply wells had been removed from service for more than 20 years. Viewed synoptically, the maps in Figure S4 illustrate a progression in the areal distribution of TCE by model layer at the HPIA from the early onset of pumping (January 1951) to substantial impact of TCE on water-supply wells (January 1968 and November 1984), to dilution and reduction in the TCE concentration at the end of the historical reconstruction simulation (June 2008) because of the cessation of pumping of historically contaminated HPIA water-supply wells. Similar areal maps were constructed for benzene migration within the HPIA and are described and provided in [1,36].

The areal distributions of reconstructed TCE concentrations for model layers 1, 3, and 5 within the HPLF for four periods—January 1968, June 1978, November 1984, and June 2008—are shown in Figure S5. Model layers 1, 3, and 5 represent major water-bearing units in the study area and are correlated with the Brewster Boulevard aquifer system, the Tarawa Terrace aquifer, and the Upper Castle Hayne aquifer, respectively [1] (Table A11). The TCE source occurring within the fate and transport model subdomain representing the HPLF was assigned to model layers 1–7. Viewed synoptically, the maps in Figure S5 illustrate a progression in the vertical (by model layer) and areal distribution of TCE within the HPLF area. January 1968 coincides with the start of the core period for the epidemiological studies, but a time prior to the onset of pumping at well HP-651. By January 1968, simulated TCE contamination within the HPLF area had migrated vertically downward to the Upper Castle Hayne aquifer, corresponding to model layer 5. June 1978 and November 1984 represent periods of substantial impact of water-supply well HP-651 on groundwater flow and the migration of TCE within the HPLF area. In the model, water-supply well HP-651 is pumping 100% from the Upper Castle Hayne aquifer (layer 5), and this effect is seen by the large cone of depression centered at, and migration of TCE to, well HP-651 during June 1978 and November 1984. Reduction in TCE concentrations began when well HP-651 was taken out of service during February 1985 [10,11] (CLW 4913). By June 2008, a shift in the simulated center of mass of the TCE plume in a northwesterly direction from well HP-651 is clearly seen in Figure S5. This shift in the center of mass of TCE is primarily caused by the influence of Wallace Creek (Figure S3b) on local groundwater flow and is more pronounced in model layers 1 and 3. Note, remediation extraction well operations began during October 1996 and pumped from model layer 5 [9] (pp. C46–C51). Similar areal maps were constructed for PCE migration within the HPLF area and are described and provided in [1,36].

### 3.2. Reconstructed Drinking-Water Concentrations

**Figure 4:**
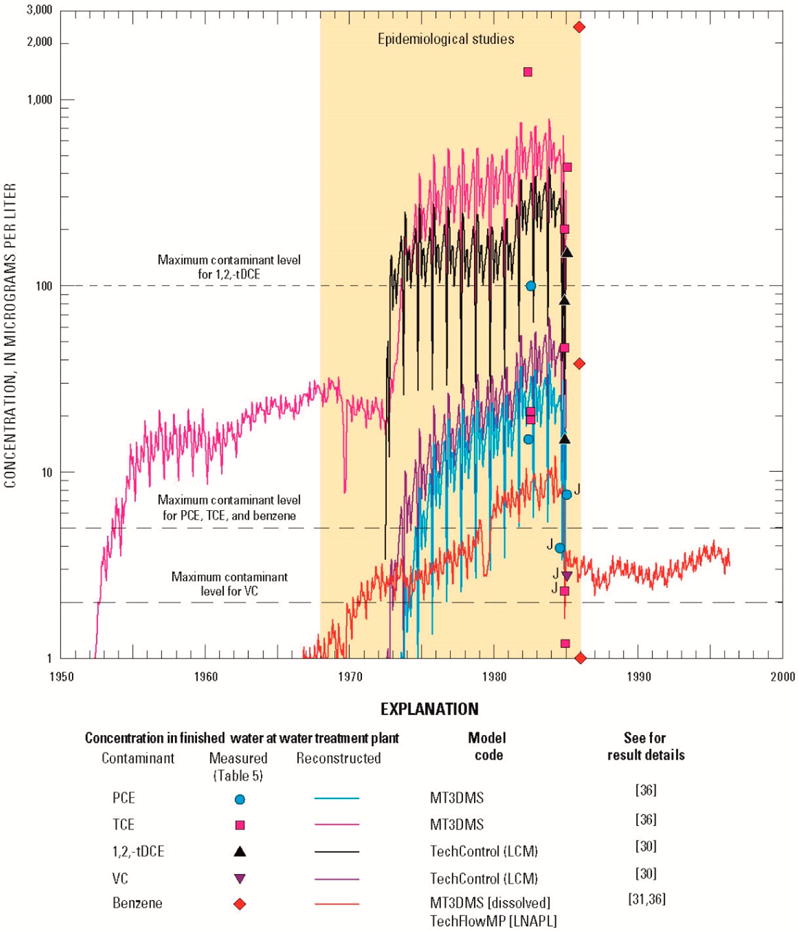
Reconstructed (simulated) drinking-water and measured concentrations of tetrachloroethylene (PCE), trichloroethylene (TCE), *trans*-1,2-dichloroethylene (1,2-tDCE), vinyl chloride (VC), and benzene at the Hadnot Point water treatment plant, U.S. Marine Corps Base Camp Lejeune, North Carolina. (See [1] for a listing of monthly mean drinking-water roncentrations; J, estimated; LCM, linear control model, LNAPL, light nonaqueous phase liquid.)

Using reconstructed (simulated) water-supply well concentrations, monthly mean concentrations of TCE were estimated for drinking water at the Hadnot Point WTP. In addition, monthly mean concentrations for PCE, 1,2-tDCE, VC, and benzene also were estimated using the approaches and methods described herein. Reconstructed monthly mean concentrations of VOCs in drinking water delivered by the Hadnot Point WTP and measured concentrations in drinking water at the WTP are shown in Figure 4 along with the current MCL for each contaminant. Measured concentrations also are listed in Table 2. Monthly reconstructed concentrations at the Hadnot Point WTP for the entire historical period (1942–2008) are provided in [1] (Appendix A7). Of note in Figure 4 is the effect of the contribution of TCE-contaminated groundwater when pumping began at water-supply well HP-651 (July 1972). TCE concentrations in drinking water at the Hadnot Point WTP ranged from about 10 to 30 μg/L for the period 1955–1972, prior to the onset of pumping from water-supply well HP-651. Subsequent to the onset of pumping of water-supply well HP-651 during July 1972, simulated drinking water contaminant concentrations increased to a maximum computed value of 783 μg/L during November 1983. It is also important to note that each VOC shown in Figure 4 was assumed to be an independent contaminant with no chemical mixing or degradation by-products. Given the limited number of measured drinking-water concentration data and their substantial variations, there is reasonable agreement between measured drinking-water concentrations and historical reconstruction results for the Hadnot Point WTP.

**Figure 5:**
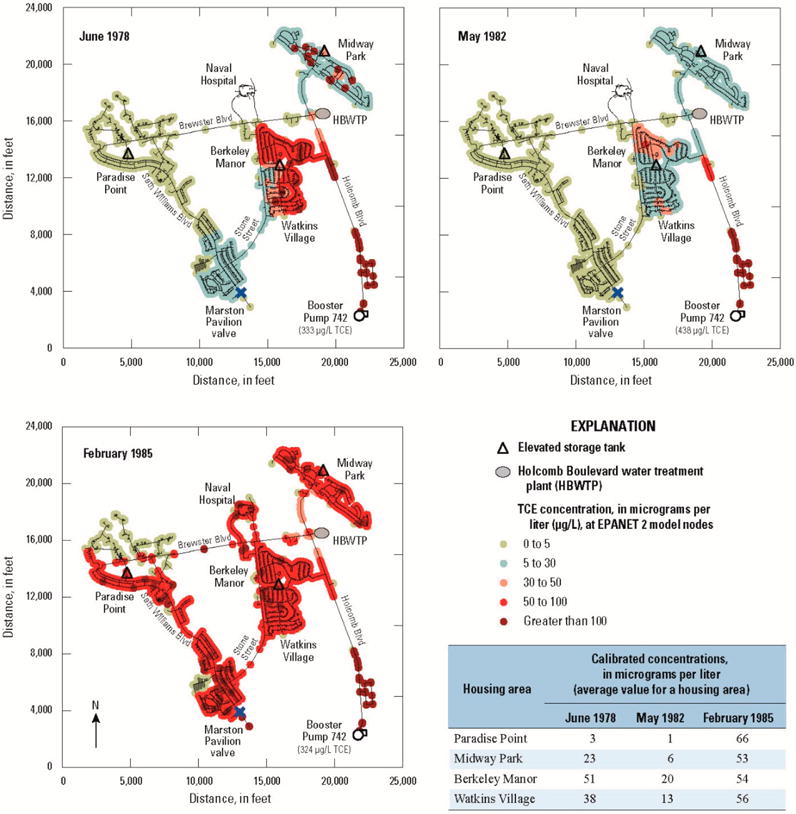
Reconstructed (simulated) distribution of trichloroethylene (TCE) contamination within the Holcomb Boulevard water treatment plant service area resulting from supply of contaminated Hadnot Point drinking water, June 1978, May 1982, and February 1985 (from [1,12]; 1 ft = 0.3048. m).

Spatial distributions of TCE levels within Holcomb Boulevard housing areas for three time periods—June 1978, May 1982, and February 1985—are shown in Figure 5. These historical reconstruction results were obtained using the EPANET 2 water-distribution system model for interconnection events. The Holcomb Boulevard reconstructed drinking-water mean TCE concentrations for the Berkeley Manor and Watkins Village housing areas during June 1978 are 51 μg/L and 38 μg/L, respectively. For May 1982, the Berkeley Manor and Watkins Village housing areas show reconstructed mean TCE concentrations of 20 μg/L and 13 μg/L, respectively. During the 8-day period of 28 January–4 February 1985 (represented by the February 1985 map in Figure 5), when the Holcomb Boulevard WTP was shut down, the reconstructed mean TCE concentrations in all housing areas exceeded 50 μg/L with the exception of the northernmost extent of Paradise Point and a small area to the north of the Marston Pavilion valve (the current MCL for TCE in drinking water is 5 μg/L). Overall, during intermittent transfers of contaminated Hadnot Point drinking water, the Paradise Point family housing area shows the lowest reconstructed mean TCE concentrations, whereas Berkeley Manor followed by Watkins Village show the greatest reconstructed mean TCE concentrations (except for the pipeline that directly connects booster pump 742 to the Holcomb Boulevard water-distribution system along Holcomb Boulevard). Spatial distribution maps for the other contaminants of concern (similar to Figure 5) are provided in [12]. Reconstructed concentrations for the other contaminants of concern (PCE, 1,2-tDCE, VC, and benzene) rarely equaled or exceeded their current MCLs during interconnection periods of interest to the ATSDR health studies.

In summary, historical reconstruction of drinking-water contaminant concentrations at the Hadnot Point WTP estimated that TCE concentrations reached a maximum value of 783 μg/L compared to a measured one-time maximum value of 1400 μg/L during the period August 1953–December 1984 (Table 2, Figure 4). The Hadnot Point WTP also provided contaminated drinking water to the Holcomb Boulevard housing area continuously prior to June 1972, when the Holcomb Boulevard WTP came on line (maximum reconstructed TCE concentration of 32 μg/L) and intermittently during the period June 1972–February 1985, with an estimated maximum reconstructed TCE concentration of 66 μg/L (Figure 5).

### 3.3. Sensitivity and Uncertainty Analyses

Best modeling practice requires that evaluations be conducted to ascertain confidence in models and model results by assessing parameter sensitivity, variability, and uncertainty associated with the modeling process and with the outcomes attributed to models [43,50]. There are numerous methods for characterizing a model’s sensitivity and uncertainty based on variations of calibrated parameter values [43,50–53]. These methods are generally classified into two groups. (1) sensitivity analysis, wherein calibrated model parameter values are varied either manually or through some automated method; and (2) probabilistic uncertainty analysis, wherein probabilistic methods are used to characterize and quantify the input and output parameter variation and uncertainty. Substantial numbers of sensitivity analyses (using a one-at-a-time method) and uncertainly analyses (using Monte Carlo simulation) were conducted as part of this study (e.g., see Table S4). Owing to brevity, these analyses arid detailed results are not presented herein; readers are referred to [1,4] for specific details and results. Based on these analyses, for the Tarawa Terrace study area, reconstructed drinking-water concentrations of PCE ranged by a factor of about 3 or less [4] (Figure A26). For the Hadnot Point-Holcomb Boulevard study area, reconstructed drinking-water concentrations of TCE ranged by a factor of about 10 or less [1] (Figure A41).

## 4. Discussion

**Figure 6:**
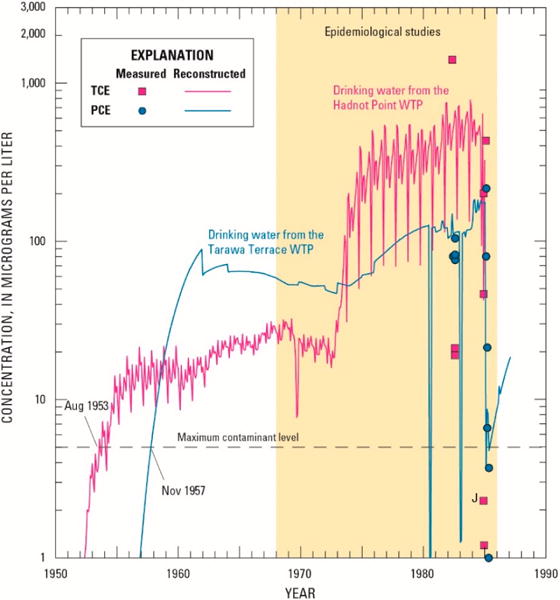
Reconstructed (simulated) drinking-water and measured concentrations of tetrachloroethylene (PCE) and trichloroethylene (TCE) at the Tarawa Terrace and Hadnot Point water treatment plants, U.S. Marine Corps Base Camp Lejeune, North Carolina (from [1,4,5]).

ATSDR conducted and completed a series of epidemiological studies to evaluate the potential for health effects from exposures to VOCs (PCE, TCE, 1,2-tDCE, VC, and benzene) in drinking water at USMCB Camp Lejeune, North Carolina, which were recently published [23–27]. These health studies required knowledge of contaminant concentrations in drinking water—at monthly intervals—delivered to family housing, enlisted personnel barracks, and workplaces within the study area. The historical reconstruction process, which included information and data mining activities and water-modeling methods, was used to quantify estimates of monthly mean contaminant-specific concentrations. Results obtained from the historical reconstruction process, water-modeling methods, and base-housing records were used in the aforementioned epidemiological studies to estimate the level and duration of exposures. Based on data, analyses, interpretations, model calibrations, and sensitivity and uncertainty analyses, the historical reconstruction process provides evidence that drinking-water TCE concentrations at the Hadnot Print WTP substentially exceeded its MCL (5 μg/L) during the periods assessed in the ATSDR epidemiological studies (Figure 6). It is most likely that in Hadnot Point drinking water TCE first exceeded its current MCL during; August 1953, but this exceedance could have been as early as November 1948 if releases of TCE to the subsurface began during or immedintely following the onset of constructian (1941/1942) of USMCB Camp Lejeune. Drinking-water contaminated with PCE exceeded the MCL for PCE (5 μg/L) du ring; the period 1975–1985 for the Hadnot Point study area and during the period 1957–1987 for the Tarawa Terrace study area (Figure 6); 1,2-tDCE, and VC also exceeded their respective MCLs (100 μg/L and 2 μg/L, respectively) during the period 1975–1985 (Figure 4). Although subatantial volumes of fuel were lost due to leakage to the subsurface during a period of about 40 years (range of 0.9 to 1.6 million gallone (3400 to 6100 m^3^) [1,31]), benzene concentrations in drinking water only slightly exceeded the MCL (5 μg/L) during the period 1980–1985 (Figure 4). Within the Holcomb Boulevard housing area, except for the 8-day period of 28 January 28–4 February 1985, when the Holcomb Boulevard WTP was out of service, only TCE routinely exceeded its MCL during intermittent periods of connection with the Hadnot Point water-distribution system (Figure5).

ATSDR has completed five epidemiological studies using the monthly mean drinking-water concentration estimates derived by the historical reconstruction process. These studies were: (1) a birth defects and childhood cancer (case-control) study [23]; (2) an adverse birth outcome (cross-sectional) study [24]; (3) a male breast cancer (case-control) study [25]; (4) a mortality study of Marines and Navy personnel (retrospective cohort study) [26]; and (5) a mortality study of civilian employees (a retrospective cohort study) at USMB Camp Lejeune [27]. Methods, analyses and results specific to each study are provided in [23–27]. The ATSDR epidemiological studies would not have been able to evaluate exposure-response relationships without the monthly mean drinking-water concentrations produced by the historical reconstruction process. Additionally, the monthly mean concentrations allowed for trimester-specific exposures to be calculated for the studies of adverse birth outcomes, including specific birth defects, and childhood hematopoietic cancers at Camp Lejeune. The ability to evaluate chemical-specific associations and exposure-response trends, rather than simply comparing exposed to unexposed, greatly enhanced the impact of these studies and the evidence they provided.

## 5. Conclusions

Given the lack and substantial limitation of historical data, multiple lines of evidence and multiple methods of analyses were used to derive water-modeling results obtained using the historical reconstruction process. These included substantial efforts in information gathering and data mining, water-modeling methods, and sensitivity and probabilistic analyses. These results provide reasonable to good agreement between measured drinking-water concentrations and reconstructed results. The results presented herein allowed epidemiologists to categorize exposure into several categories rather than relying on crude classification estimates of exposed versus unexposed populations for the epidemiological studies at USMCB Camp Lejeune, North Carolina. The ability to evaluate exposure-response trends, rather than simply comparing exposed to unexposed, greatly enhanced the impact of these studies and the evidence they provided.

## Supplementary Material

Supplemental Info

Supplental Info

## Figures and Tables

**Table 1:** Analyses and simulation tools used to reconstruct historical drinking-water concentrations at U.S. Marine Corps Base Camp Lejeune, North Carolina.

Analysis	Description	Analysis Type and Simulation Tool	Reference
Geohydrologic framework	Detailed analyses of well and geohydrologic data used to develop framework of the Brewster Boulevard and Castle Hayne aquifer systems and Tarawa Terrace aquifer	Data analysis and interpretation	[32]
Water-level analyses and groundwater flow	Characterizations of water-level data and groundwater flow	Data analysis and interpretation	[33]
Predevelopment groundwater flow	Steady-state, three-dimensional groundwater flow, occurring prior to initiation of water-supply well activities (1942) using a grid of uniform cells of 300 ft × 300 ft (91 m × 91m)	Simulation using MODFLOW-2005	[34,35]
Historical water-supply well operations	Documenting water-supply well capacities, histories, and reconstructing operating schedules on a monthly basis for the period 1942–2008 (e.g., Figure S1)	Data analysis, interpretation, and simulation using TechWellOP	[10,29]
Transient groundwater flow	Unsteady-state, three-dimensional groundwater flow occurring primarily because of the initiation and continued operation of water-supply wells (July 1942–June 2008), using a variably-spaced grid ranging in area from 300 ft × 300 ft (91 m × 91 m) to 50 ft × 50 ft (15 m × 15 m) in the HPIA and HPLF model subdomain areas (e.g., Figure S2)	Simulation using MODFLOW-2005	[34,35]
Properties of VOCs in groundwater	Properties of degradation pathways of common organic compounds in groundwater	Literature survey	[2]
Occurrence of selected contaminants in groundwater	Description and summaries of groundwater contaminants of selected VOCs and BTEX components at IRP and AST/UST sites; listing of water-supply and monitor well location and construction data	Data analysis	[9,13]
Computation of mass for PCE, TCE, and benzene	Estimates of mass (volume) of TCE, PCE, and benzene in groundwater using field data and a variety of analytical and numerical techniques	Site investigation data, GIS spatial analyses, LNAPL volume analyses (TechNAPLVol)	[9,13,31,36]
Fate and transport of TCE, PCE, and benzene	Simulation of the fate and migration of TCE and dissolved benzene from sources in the HPIA; simulation of the fate and migration of PCE from the HPLF;	Simulation using MT3DMS-5.3	[36–38]
Fate and transport of benzene (LNAPL)	Simulation of the fate and migration of benzene as an LNAPL from sources at the Handot Point fuel farm in the HPIA;	Simulation using TechFlowMP	[31,39–41]
Concentrations of PCE, TCE, 1,2-tDCE, and VC in a water-supply well	Reconstructing concentrations of PCE, TCE, 1,2-tDCE, and VC in water-supply HP-651 (HPLF)) using a linear control model methodology	Simulation using TechControl	[30]
TCE, PCE, 1,2-tDCE, VC, and benzene in WTP drinking water	Computations of concentrations of TCE, PCE, 1,2-tDCE, VC, and benzene in drinking water from the Hadnot Point WTP using results from fate and transport and linear control model simulations	Materials mass balance model using principles of conservation of mass and continuity—algebraic	[36,42]
Parameter uncertainty and variability	Assessment of parameter sensitivity and uncertainty associated with model simulations of groundwater flow, fate and transport, and water distribution	One-at-a-time sensitivity analysis (OAT), Monte Carlo (MC) simulation using Latin hypercube sampling (LHS), and MC simulation	[12,35,36,43]
Intermittent pump operation for transfer of drinking water	Probabilistic analysis of the occurrence of pump operations during the period 1972–1985 for transferring Hadnot Point drinking water to Holcomb Boulevard housing areas	Probabilistic Markov analysis using TechMarkovChain	[12,44]
Distribution of TCE, PCE, 1,2-tDCE, VC, and benzene throughout the Holcomb Boulevard housing areas	Simulation of hydraulics and water-quality in the water-distribution system serving the Holcomb Boulevard housing areas, 1972–1985; intermittent pump operations estimated using data and Markov analysis	Simulation using EPANET 2	[12,45]

**Table 2:** Selected measured and reconstructed (simulated) concentrations of tetrachloroethylene (PCE), trichloroethylene (TCE), *trans*-1,2-dichloroethylene (1,2-tDCE), vinyl chloride (VC), and benzene at the Hadnot Point water treatment plant, U.S. Marine Corps Base Camp Lejeune, North Carolina.

Contaminant	Measured Data^1^		Reconstructed (Simulated)^2^		Reconstructed (Maximum Value)^2^	

	Sample Date	Concentration, in μg/L	Simulation Date	Concentration, in μg/L	Simulation Date	Concentration, in μg/L
PCE	27 May 1982^3^	15	May 1982	21	November 1983	39
	27 July 1982^4^	100	July 1982	27		
	4 December 1984^6^	3.9 J	November 1984	31		
	5 February 1985^7^	7.5 J	January 1985	16		

TCE	27 May 1982^3^	1400	May 1982	438	November 1983	773
	27 July 1982^5^	19	August 1982	670		
	27 July 1982^6^	21	August 1982	670		
	4 December 1984^5^	46	November 1984	639		
	4 December 1984^6^	200	November 1984	639		
	12 December 1984^6^	2.3 J	December 1984	43		
	19 December 1984	1.2	December 1984	43		
	5 February 1985^7^	429	January 1985	324		

1,2-tDCE	4 December 1984^6^	83	November 1984	358	November 1983	435
	4 December 1984^5^	15	December 1984	26		
	12 December 1984^6^	23 J	December 1984	26		
	5 February 1985^7^	150	January 1985	163		

VC	5 February 1985^7^	2.9 J	January 1985	31	November 1983	67

Benzene	19 November 1985^7^^,^^8^^,^^9^	2500	November 1985	3	April 1984	12
	10 December 1985^7^	38	December 1985	3		
	18 December 1985^7^	1	December 1985	3		

Notes:

1 Data from [9] (Tables C11 and C12);

2 Simulation results represent the last day of each month (e.g., 31 May); results reported for simulation month nearest the sample date; refer to [1] (Appendix A7) for complete listing of reconstructed treated-water concentrations;

3 Water sample collected at Building NH-1; data reported as unreliable;

4 Water sample collected at Building FC-530;

5 Untreated water;

6 Treated water;

7 Treatment status unknown;

8 Laboratory analysis noted with: “Sample appears to have been contaminated with benzene, toluene, and methyl chloride” [49];

9 Data noted with: “Not Representative” [11] (CLW 1356); J, estimated value.

## Abbreviations

AST/UST: above ground storage tank/underground storage tank
ATSDR: Agency for Toxic Substances and Disease Registry
BTEX: benzene, toluene, ethylbenzene, and xylene
C: source concentration
CLW: Camp Lejeune water document
EPANET 2: water-distribution system model developed by the USEPA
EPS: extended period simulation
ft: foot or feet
GIS: geographical information system
GT-MESL: Georgia Institute of Technology, Multimedia Environmental Simulations Laboratory
HPIA: Hadnot Point Industrial Area
HPFF: Hadnot Point fuel farm
HPLF: Hadnot Point landfill
I: infiltration
IRP: Installation Restoration Program of the U.S. Department of the Navy
J (in data tables): estimated value
KD: distribution coefficient
km: kilometer or kilometers
K_xx_, K_zz_: horizontal and vetical hydraulic conductivity
LCM: linear control model
LNAPL: light nonaqueous-phase liquid
m: meter or meters
MCL: maximum contaminant level
mg/L: milligram per liter
mi: mile or miles
MODFLOW: modular, three-dimensional groundwater flow model developed by the United States Geological Survey
n_E_: porosity
NPL: National Priorities List
PCE: tetrachloroethylene
Q_monthly_: historical monthly pumping
SI: supplemental information
TCE: trichloroethylene
USEPA: United States Environmental Protection Agency
USMCB: United States Marine Corps Base
VC: vinyl chloride
VOC: volatile organic compound
WTP: water treatment plant
α_L_, α_T_, α_V_: longitudinal, transverse, and vertical dispersivity
Δt: time-step size
Δx, Δy: finite-difference cell size
λ: biodegradation rate
ρ_b_: bulk density
μg/L: micrograms per liter
1,2-tDCE: trans-1,2-dichloroethylene
