# Supplementary material for: Reconstructing Historical VOC Concentrations in Drinking Water for Epidemiological Studies at a U.S. Military Base: Summary of Results
Source: Water (Basel). Author manuscript; Available in PMC 2017 Sep 1. (PMC5580837; doi:10.3390/w8100449)
Supplement: Supplemental Info [file NIHMS897944-supplement-Supplemental_Info.pdf]

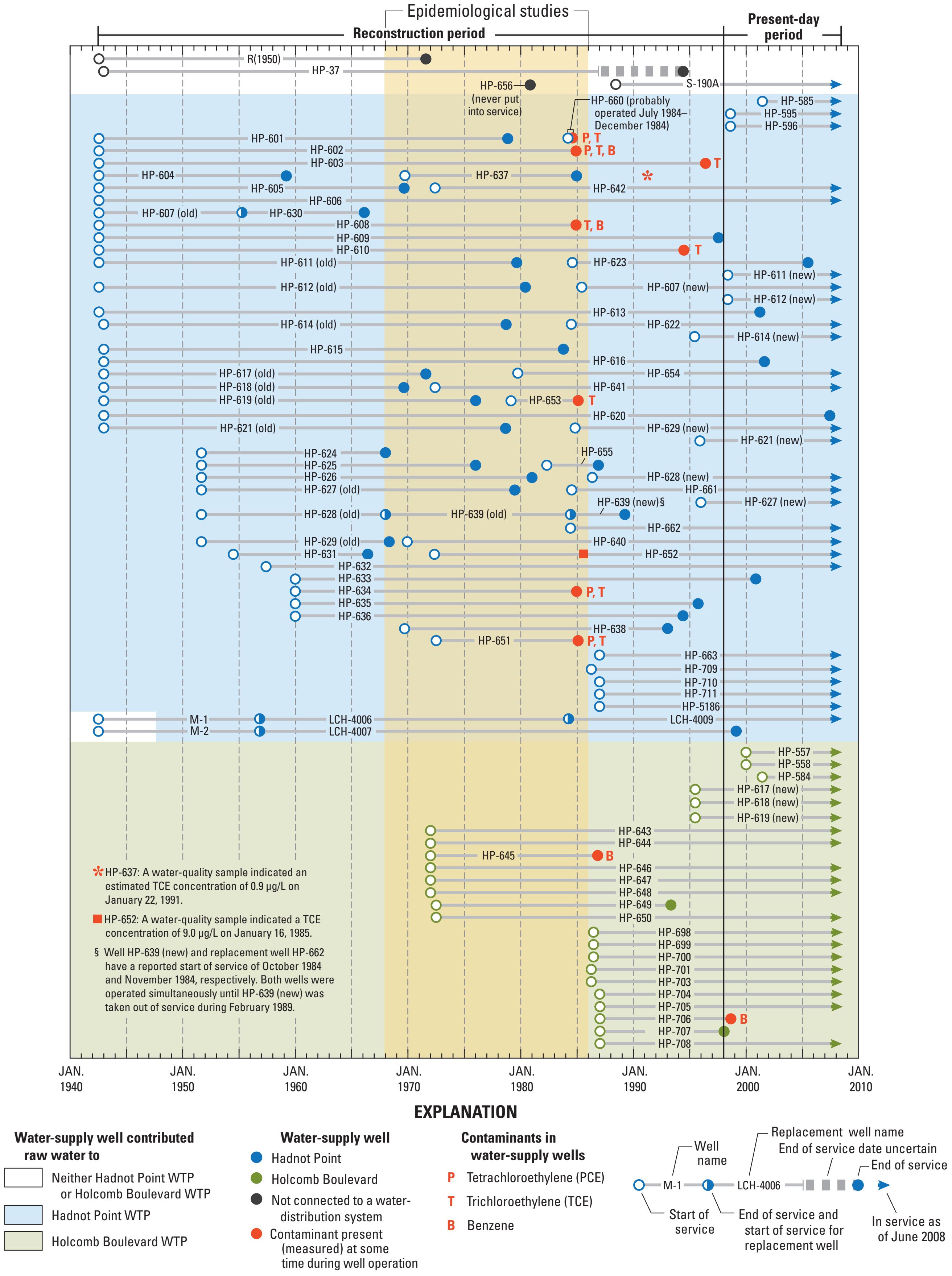

Figure S-1. Maslia et al. 2016

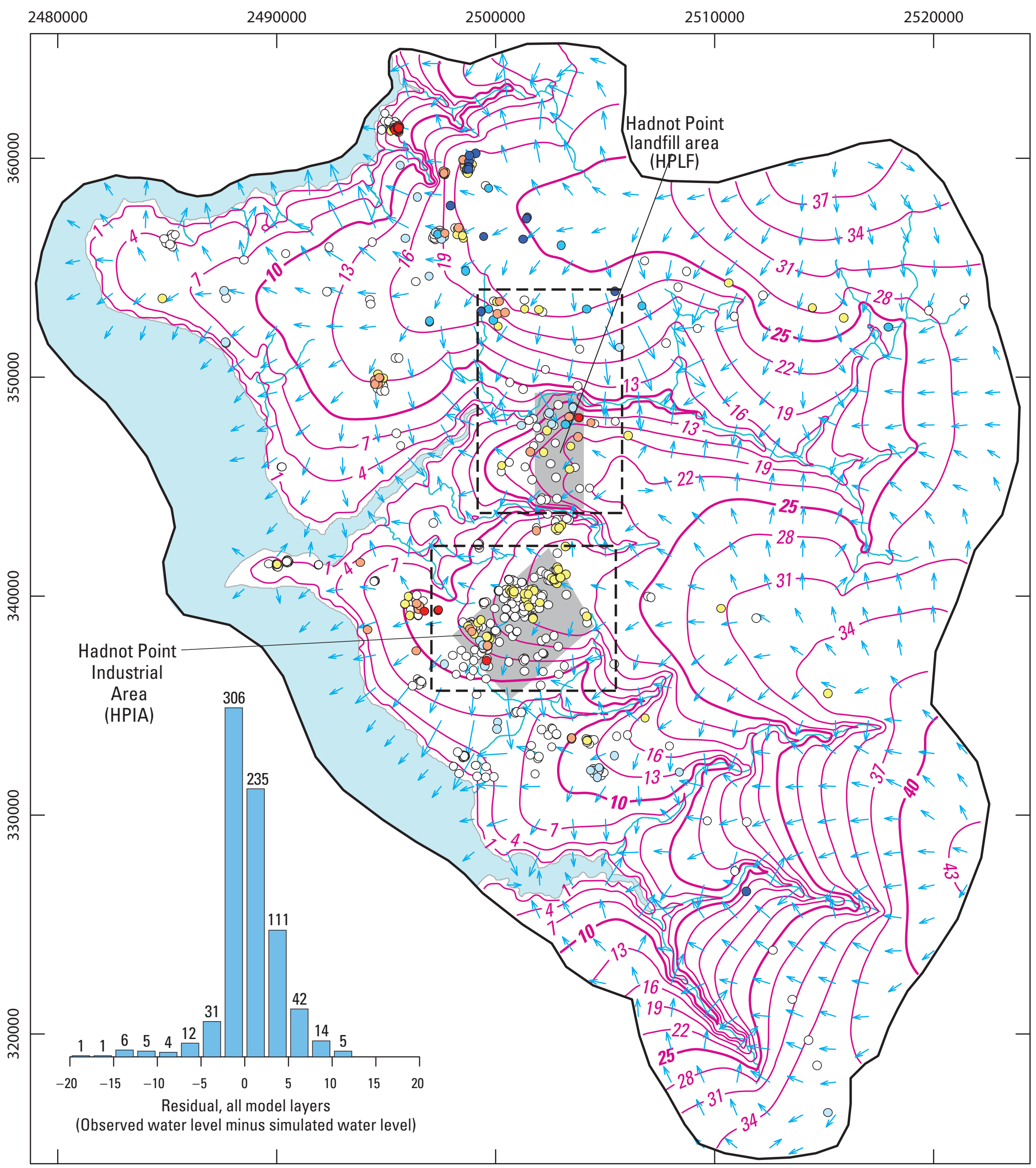

Base from U.S. Marine Corps and U.S. Geological Survey digital data files
Map coordinates are North Carolina State Plane coordinates
North American Datum of 1983

0 1 2 MILES

0 1 2 KILOMETERS

EXPLANATION

Active model domain

Contaminant fate and transport model subdomain

Simulation results

10 — Potentiometric contour—Contour interval 3 feet. Datum is NGVD 29

Direction of groundwater flow

Residual

- >7.5
- 5 to 7.5
- 2.5 to 5
- 2.5 to −2.5
- −2.5 to −5
- −5 to −7.5
- <−7.5

Residual statistics, all model layers

Number of terms	773
Minimum	−17.5 feet
Maximum	11.5 feet
Average residual	0.5 feet
Standard deviation	3.34
Root-mean square residual	3.39 feet

Figure S-2. Maslia et al. 2016

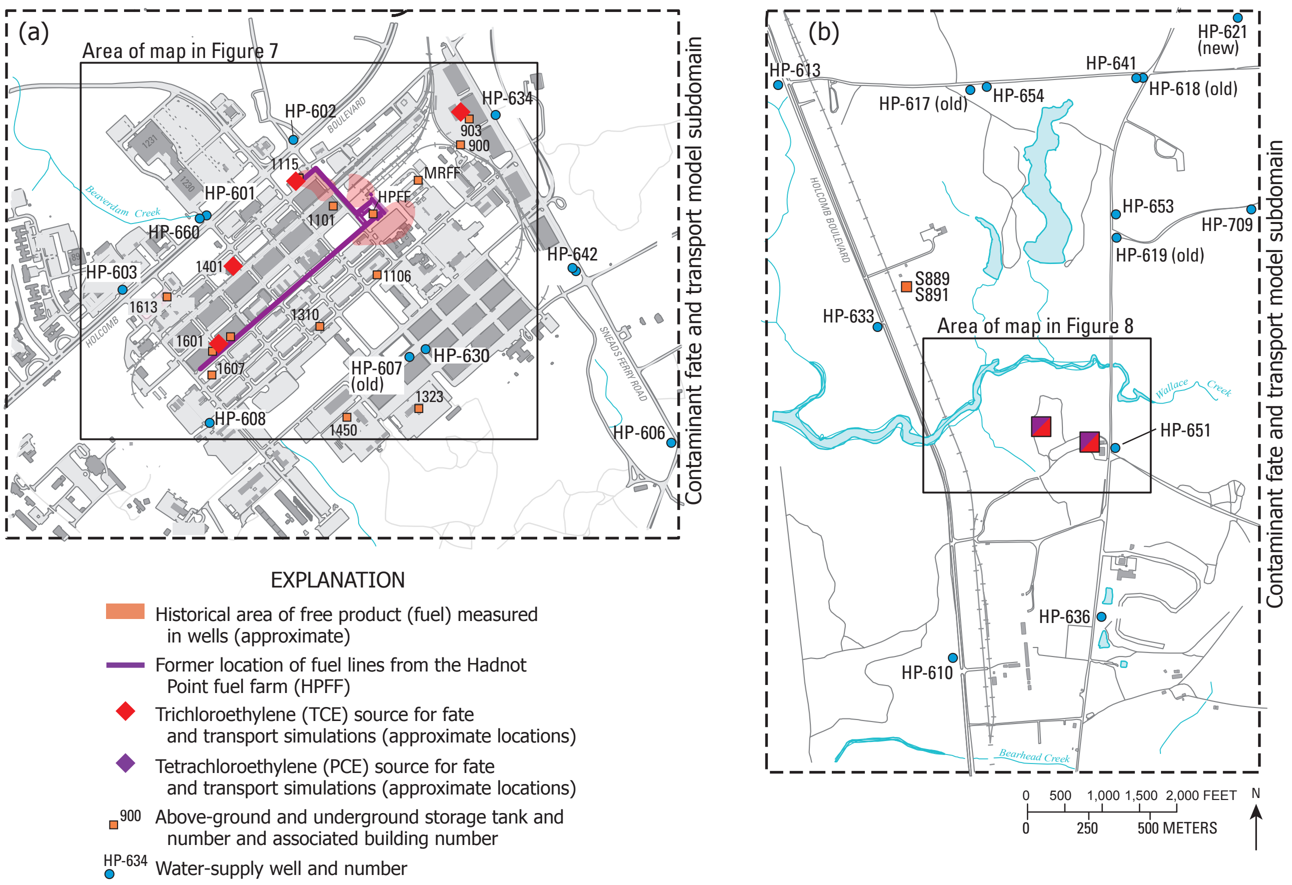

Figure S-3. Maslia et al. 2016

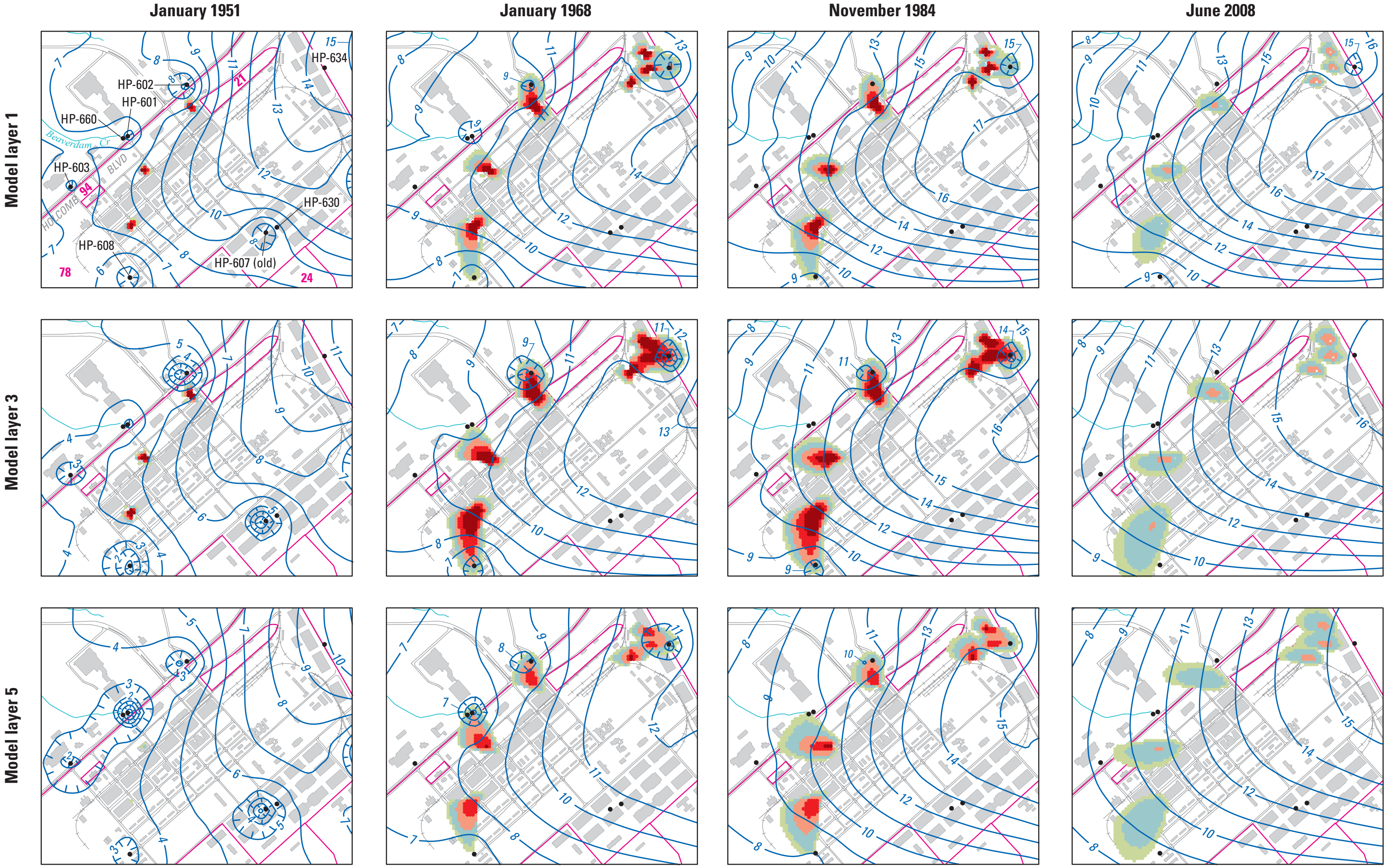

Base modifed from U.S. Marine Corps digital data files

0 1,000 2,000 FEET

0 250 500 METERS

EXPLANATION

78 **Installation Restoration Program site boundary and number**

HP-634 **Water-supply well and number**

8 **Simulated potentiometric contour**—Shows simulated potentiometric surface. Hachures indicate depression. Contour interval 1 foot. Datum is National Geodetic Vertical Datum of 1929

TCE concentration, in micrograms per liter

- 1 to 5
- Greater than 5 to 50
- Greater than 50 to 500
- Greater than 500 to 5,000
- Greater than 5,000 to 1,000,000

Figure S-4. Maslia et al. 2016

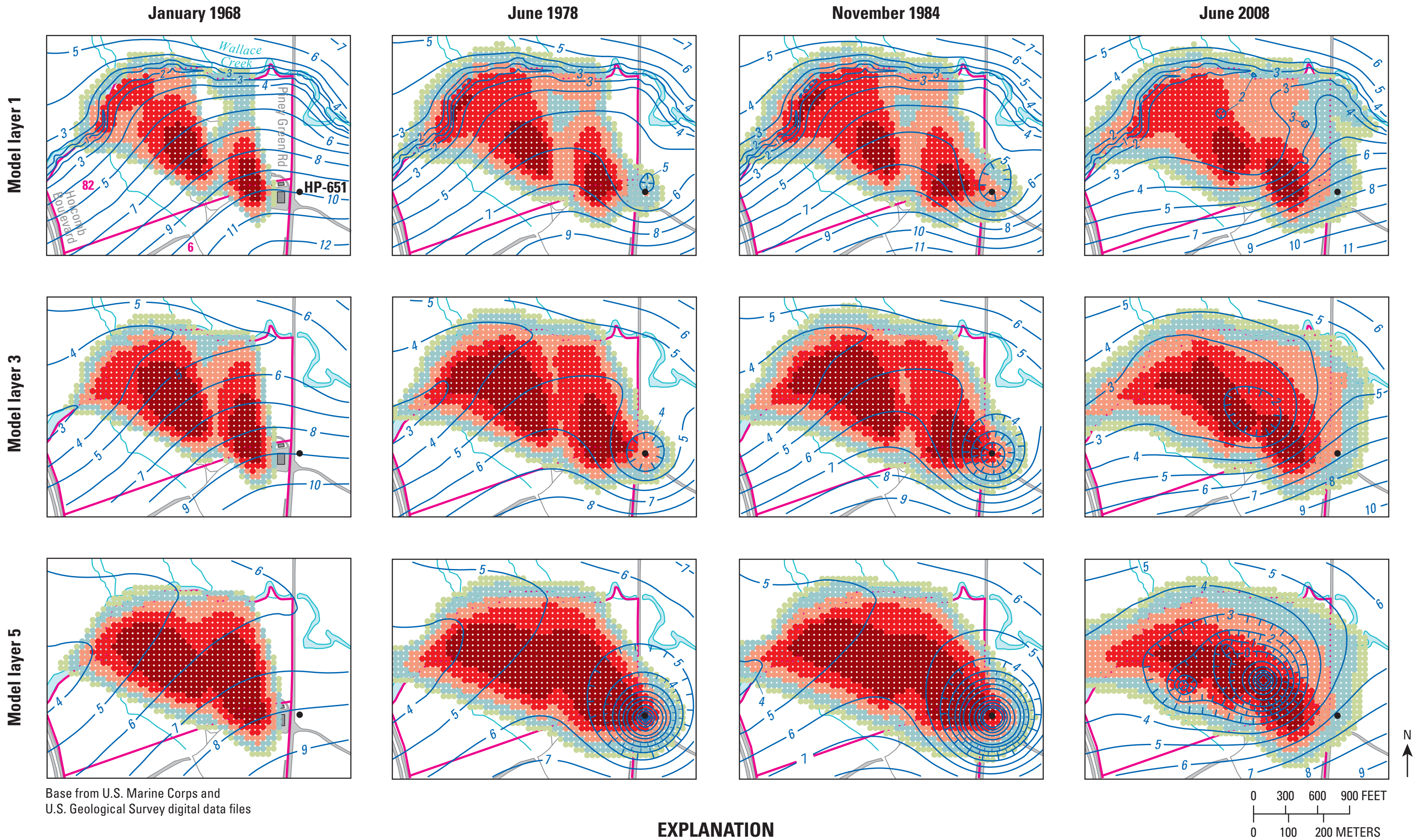

Base from U.S. Marine Corps and
U.S. Geological Survey digital data files

EXPLANATION

82 **Installation Restoration Program site boundary and number**

HP-651 **Pumping water-supply well and identification**

8 **Simulated potentiometric contour**—Shows simulated potentiometric surface. Hachures indicate depression. Contour interval, in feet, is variable. Datum is National Geodetic Vertical Datum of 1929

TCE concentration, in micrograms per liter

- 1 to 5
- Greater than 5 to 50
- Greater than 50 to 500
- Greater than 500 to 5,000
- Greater than 5,000 to 400,000

Figure S-5. Maslia et al. 2016

Table S-1. Chronology of selected evens related to water supply and environmental contamination at U.S. Marine Corps Base Camp Lejeune, North Carolina, and vicinity. #,*

Event	Date or approximate date
Hadnot Point water treatment plant (WTP) comes on line	1941–42
Tarawa Terrace WTP comes on line	1952–53
Holcomb Boulevard WTP comes on line	June 1972
Several Tarawa Terrace and Hadnot Point water-supply wells shut down due to documented volatile organic compound (VOC) contamination	November 1984–February 1985
Marston Pavilion interconnection valve opened and booster pump 742 continuously operated for eight days (because of shut down of Holcomb Boulevard WTP) to augment Holcomb Boulevard drinking-water supply with contaminated Hadnot Point drinking water	January 27–February 4, 1985
Holcomb Boulevard WTP expanded to provide water to Tarawa Terrace and Camp Johnson water-distribution system areas	1987
Tarawa Terrace WTP and remaining operating supply wells shut down and taken out of service	March 1987
ABC One-Hour Cleaners placed on the USEPA's National Priorities List (NPL) of contaminated sites	March 1989
USMCB Camp Lejeune placed on the USEPA's NPL of contaminated sites	October 1989

#Refer to [1,4] for details; *see Figure 1 for location of water-supply areas.

Table S-2. Calibrated model parameter values used to simulate groundwater flow and contaminant fate and transport at U.S. Marine Corps Base Camp Lejeune, North Carolina.[¶]

Model Parameter[+]	Model layer number[#] 1	2	3	4	5	6	7
**Pre-development (steady-state) groundwater flow model, conditions prior to 1942 — Uniform grid (300 ft x 300 ft cells)[§]**							
Horizontal hydraulic conductivity, K_{xx} (ft/d)	0.5–46.8	1.0–20.0	1.0–50.0	1.0–35.0	2.3–50.0	1.0–20.0	20.0
Ratio of vertical to horizontal hydraulic conductivity, K_{zz}/K_{xx}	1:10	1:15	1:10	1:15	1:10	1:15	1:10
Infiltration (recharge), $I_{i,j}$ (in./yr)	2.5–22.0	—	—	—	—	—	—
**Transient groundwater flow model, January 1942–June2008 — Variable-spaced grid (300 ft x 300 ft – 50 ft x 50 ft cells)[§]**							
Specific yield, S_y	0.05	—	—	—	—	—	—
Specific storage, S_s[++] (1/ft)	—	$1.3\text{x}10^{-5}$–$1.9\text{x}10^{-4}$	$4.3\text{x}10^{-6}$–$3.6\text{x}10^{-5}$	$1.0\text{x}10^{-5}$–$3.8\text{x}10^{-5}$	$4.0\text{x}10^{-6}$–$8.3\text{x}10^{-6}$	$1.4\text{x}10^{-5}$–$3.6\text{x}10^{-5}$	$3.4\text{x}10^{-6}$–$7.7\text{x}10^{-6}$
Infiltration (recharge), $I_{i,j}$ (in./yr)	Varies[##]	—	—	—	—	—	—
Pumpage, Q (ft^3/d)[§§]	Varies	Varies	Varies	Varies	Varies	Varies	Varies
**Contaminant fate and transport models, January 1942 1951–June2008 — subdomain area (50 ft x 50 ft cells)[§]**							
Distribution coefficient, K_D (ft^3/mg)[***]							
PCE	$5.0\text{x}10^{-6}$	$5.0\text{x}10^{-6}$	$5.0\text{x}10^{-6}$	$5.0\text{x}10^{-6}$	$5.0\text{x}10^{-6}$	$5.0\text{x}10^{-6}$	$5.0\text{x}10^{-6}$
TCE	$5.3\text{x}10^{-9}$	$5.3\text{x}10^{-9}$	$5.3\text{x}10^{-9}$	$5.3\text{x}10^{-9}$	$5.3\text{x}10^{-9}$	$5.3\text{x}10^{-9}$	$5.3\text{x}10^{-9}$
Benzene	$4.0\text{x}10^{-9}$	$4.0\text{x}10^{-9}$	$4.0\text{x}10^{-9}$	$4.0\text{x}10^{-9}$	$4.0\text{x}10^{-9}$	$4.0\text{x}10^{-9}$	$4.0\text{x}10^{-9}$
Bulk density, ρ_b (g/ft^3)	47,000	47,000	47,000	47,000	47,000	47,000	47,000
Effective porosity, n_E	0.2	0.2	0.2	0.2	0.2	0.2	0.2
Biodegradation, λ(d^{-1})							
HPIA (TCE)	$2.0\text{x}10^{-3}$	$2.0\text{x}10^{-3}$	$2.0\text{x}10^{-3}$	$2.0\text{x}10^{-3}$	$2.0\text{x}10^{-3}$	$2.0\text{x}10^{-3}$	$2.0\text{x}10^{-3}$
HPIA (benzene)	$1.0\text{x}10^{-4}$	$1.0\text{x}10^{-4}$	$1.0\text{x}10^{-4}$	$1.0\text{x}10^{-4}$	$1.0\text{x}10^{-4}$	$1.0\text{x}10^{-4}$	$1.0\text{x}10^{-4}$
HPLF (PCE and TCE)	$1.4\text{x}10^{-4}$	$1.4\text{x}10^{-4}$	$1.4\text{x}10^{-4}$	$1.4\text{x}10^{-4}$	$1.4\text{x}10^{-4}$	$1.4\text{x}10^{-4}$	$1.4\text{x}10^{-4}$
Effective molecular diffusion coefficient, D^* (ft^2/d)	$1.0\text{x}10^{-3}$	$1.0\text{x}10^{-3}$	$1.0\text{x}10^{-3}$	$1.0\text{x}10^{-3}$	$1.0\text{x}10^{-3}$	$1.0\text{x}10^{-3}$	$1.0\text{x}10^{-3}$

Disperssivity (ft)							
Longitudinal, α_L	25	25	25	25	25	25	25
Transverse, α_T	2.5	2.5	2.5	2.5	2.5	2.5	2.5
Vertical, α_V	0.25	0.25	0.25	0.25	0.25	0.25	0.25
Source concentration, *C* (mg/L)							
HPIA (TCE)	640	640	640	640	640	640	640
HPIA (benzene–dissolved)	1.7	—	—	—	—	—	—
HPIA (benzene–LNAPL)	17	—	—	—	—	—	—
HPLF (PCE)	42–105	33–83	27–66	18–46	6–16	0	0
HPLF (TCE)	256–384	256–384	256–384	256–384	256–384	256–384	256–384

¶Refer to [31,35,36] for details;

+Symbolic notation used to describe model parameters obtained from [34,37,38];

#See [1] for correlation between geologic and hydrogeologic units and model layers for the Hadnot Point and Holcomb Boulevard areas; refer to [32,35] for details; aquifers are designated as model layers 1, 3, 5, and 7; confining units are designated as model layers 2, 4, and 6;

§See Figure SI-2 for groundwater-flow model domain and contaminant fate and transport model subdomains;

++ Specific storage (S_s)was specified as input for MOFLOW-2005 [34]; based on cell-by-cell thicknesses, specific storage was varied to assure a constant storage coefficient (or storativity) of 4 x 10^{-4} using the equation $S = S_s \times b$, where S is the storage coefficient (dimensionles), S_s is specific storage (1/ft), and b is the cell thickness (ft);

##Transient infiltration was varied on a monthly basis using the ratio of monthly precipitation divided by average, long-term precipitation; [35] for details;

§§Pumpage varies by month and model cell; refer to [10,29] for details on the derivation of historical monthly water-supply well operations; refer to [35] for details pertaining to assigning monthly water-supply well pumpage to cells and model layers using the multi-node well-flow package for MODFLOW-2005;

***Refer to [36] for derivation of K_D values based on a survey reported in the scientific literature; to convert from model K_D units of ft^3/mg to units of L/kg reported in [36], multiply model K_D values by 28,381,652.21.

Table S-3. Contaminant sources, locations, and durations used for historical reconstruction of TCE, PCE, and benzene concentrations in groundwater at U.S. Marine Corps Base Camp Lejeune, North Carolina [#, ¶].

Source Location	Contaminant	Source concentration (µg/L)[+]	Number of model sources[§]	Source duration
**Hadnot Point Industrial Area (Figure SI3a)**				
Building 900 area	TCE	640	3	Jan. 1957–Dec. 1994
Building 1115	TCE	640	1	Jan. 1951–Jun. 1993
Building 1401	TCE	640	1	Jan. 1951–Dec. 1993
Building 1601	TCE	640	1	Jan. 1951–Jun. 1993
Building 1601	Benzene (dissolved)	1.7	1	Jan. 1951–Dec. 1994
Building 1613	Benzene (LNAPL)[**]	17	Multiple[**]	Jan. 1964–Jun. 2008
Hadnot Point fuel farm	Benzene (LNAPL)[**]	17	Multiple[**]	Jan. 1951–Jun. 2008
**Hadnot Point landfill area (Figure SI3b)**				
Source 1[++]	TCE	256–384[±]	2	Jan. 1948–Jun. 2008
Source 2[++]	PCE	6–105[±]	2	Jan. 1948–Jun. 2008

[#] See [1] for complete details.

[¶] All model sources are specified concentration; model simulation time is January 1942–June 2008; refer to [31,36] for details.

[+] Current maximum contaminant level (MCL) for TCE, PCE, and benzene is 5 µg/L; density (20°C): TCE, 1.464 g/cm^3; PCE, 1.623 g/cm^3; benzene, 0.876 g/cm^3 [2]; solubility in water (25°C): TCE, 1,280 mg/L; PCE, 210 mg/L; benzene, 1,780 mg/L [2].

[§] Refer to [31,36] for details.

[**] Benzene source for model is areally distributed based on LNAPL distribution; refer to [31] for details.

[++] There are no designated building numbers within the Hadnot Point landfill; location of sources based on Installation Restoration Program Site 82 history and contaminant analyses [9] and model calibration [36].

[±] Source concentration values vary by model layer, refer to [1 (Table A12),31,36] for details.

Table S-4. Types of sensitivity analyses applied to models and parameters (from [1]).

<table>
<tr><th rowspan="2"></th><th colspan="3">TYPE OF VARIATION</th></tr>
<tr><th></th><th>Physical parameters</th><th>Numerical parameters</th></tr>
<tr><td rowspan="3">TYPE OF SIMUALTION MODEL</td><td>Groundwater flow</td><td>Historical monthly pumping: $Q_{monthly}$

Input parameters/hydraulics: K_{xx}</td><td>Cell size: Δx, Δy</td></tr>
<tr><td>Contaminant fate and transport</td><td>Input parameters/fate and transport: ρ_b, n_E, α_L, K_D, λ, C

Benzene source area and release

Trichloroethylene source-release date</td><td>Cell size: Δx, Δy

Time-step size: Δt</td></tr>
<tr><td>Water distribution system*</td><td>Pipe roughness: C-factor

Storage-tank mixing</td><td>Demand factor</td></tr>
</table>

*Sensitivity analyses using water-distribution system model conducted as part of the Tarawa Terrace study area [46].
